# The non-equilibrium phase diagrams of flow-induced crystallization and melting of polyethylene

**DOI:** 10.1038/srep32968

**Published:** 2016-09-09

**Authors:** Zhen Wang, Jianzhu Ju, Junsheng Yang, Zhe Ma, Dong Liu, Kunpeng Cui, Haoran Yang, Jiarui Chang, Ningdong Huang, Liangbin Li

**Affiliations:** 1 National Synchrotron Radiation Lab, CAS Key Laboratory of Soft Matter Chemistry, University of Science and Technology of China, Hefei, China; 2 Tianjin Key Laboratory of Composite and Functional Materials, and School of Materials Science and Engineering, Tianjin University, Tianjin 300072, China

## Abstract

Combining extensional rheology with *in-situ* synchrotron ultrafast x-ray scattering, we studied flow-induced phase behaviors of polyethylene (PE) in a wide temperature range up to 240 °C. Non-equilibrium phase diagrams of crystallization and melting under flow conditions are constructed in stress-temperature space, composing of melt, non-crystalline δ, hexagonal and orthorhombic phases. The non-crystalline δ phase is demonstrated to be either a metastable transient pre-order for crystallization or a thermodynamically stable phase. Based on the non-equilibrium phase diagrams, nearly all observations in flow-induced crystallization (FIC) of PE can be well understood. The interplay of thermodynamic stabilities and kinetic competitions of the four phases creates rich kinetic pathways for FIC and diverse final structures. The non-equilibrium flow phase diagrams provide a detailed roadmap for precisely processing of PE with designed structures and properties.

Flow-induced crystallization (FIC) is a non-equilibrium thermodynamic phase transition occurring in daily industrial processing of semicrystalline polymers with an annual consumption of hundred million metric tons. Imposing an external flow can enhance nucleation^1^,^2^, induce intermediate orders^3^,^4^ and tune crystalline structure and morphology^5^,^6^,^7^,^8^, which consequently determines the properties of final products. Over the past decades, much effort has been devoted to understanding the thermodynamic and molecular mechanisms behind the assisting role of flow on crystallization^9^. However, the non-equilibrium nature of FIC is still poorly understood.

Without considering whether thermodynamic equilibrium or non-equilibrium, phase diagram is always the central issue of phase transition, as it determines the transition direction and guides the kinetic pathways. Unfortunately, no single non-equilibrium phase diagram of FIC of polymers has been constructed yet. Lacking non-equilibrium phase diagrams, current thermodynamic models of FIC mainly consider the shifting of crystallization or melting point along temperature axis^10^, but ignore the structural intermediates, which may be the essential features reflecting the non-equilibrium nature of FIC, although flow-induced different crystal modifications can be incorporated recently^11^,^12^. Are flow-induced intermediate orders kinetic states or thermodynamic phases? When and how do different orders and crystal forms appear, and compete or couple with each other? All these questions are still open.

Current studies of FIC are mainly performed at temperatures below the equilibrium freezing point, where flow enhances crystallization and crystal appeared remains stable even after the cessation of flow. Restricted within the two-phase model of classical nucleation theory, the most widely used model of FIC simply attributes the enhanced crystallization to entropic reduction of initial melt due to flow-induced chain orientation or stretch^10^. However, as a non-equilibrium driven force, flow is prone to inducing kinetically favorable intermediate orders between the initial melt and the final crystals, which may break the two-phase model and play an important role in enhancing crystallization. Reports on FIC at temperatures well above the equilibrium melting point are rather rare. Nevertheless, sufficiently strong flow may reverse the relative stabilities of different phases and induce the occurrence of crystallization as a dynamic phase transition, where crystal melts after flow is removed^13^,^14^. Because of non-equilibrium dissipative nature, here appearing and surviving of crystal depend critically on the flow strength. Dynamic phase transitions due to reversed thermodynamic stabilities and kinetic pathways with transient intermediate orders are two essential features reflecting the non-equilibrium nature of FIC.

In this article, the dynamics and kinetics of crystallization/melting of polyethylene (PE) under extension flow are studied with *in-situ* synchrotron ultrafast x-ray scattering. Analogue to the non-equilibrium phase diagrams of flow-induced phase separation^15^ and jamming-unjamming in colloid system^16^, we construct the non-equilibrium phase diagrams for crystallization/melting of PE in stress-temperature space. Stress is selected as the non-equilibrium parameter as it reflects the deformations of molecular chain and crystal, which couples the effects of both strain and strain rate. Four phase regions of melt, non-crystalline δ phase, hexagonal and orthorhombic crystals are included. The non-crystalline δ is demonstrated to be a thermodynamic phase rather than a kinetic state, which can be either metastable or stable in stress-temperature space. With our non-equilibrium crystallization/melting flow diagrams, the observed FIC phenomena in PE melt can be well understood.

## Results

### Multistep kinetic pathways of FIC

**Figure 1:**
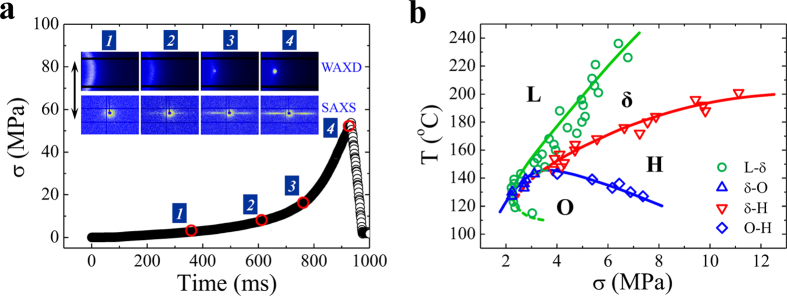
*In-situ* WAXD/SAXS characterization and crystallization phase diagram. (**a**) Representative true stress (σ)-time curve during extension with strain rate of 3 s^−1^ at 172 °C. The inserted images display selected 2D WAXD and SAXS patterns collected at the numbered points. Sample fracture happens at time of 930 ms indicated by sudden reduction of stress. The extensional direction is vertical as shown by the arrow. (**b**) The non-equilibrium crystallization phase diagram of crosslinked PE in stress-temperature space. Four phases of melt (L), non-crystalline shish (δ), H-crystal (H) and O-crystal (O) are included.

Crosslinked PE with a gel fraction of 43.5% was employed as the model sample due to its accessibility of high stress without fracture even at temperature far above the melting point. It was prepared by γ-ray radiation on a commercial PE with a quiescent equilibrium melting point of 141.4 °C^17^. It should be mentioned that we have done a similar research on the non-crosslinked PE and verified the conclusions in this paper (Supplementary Figs S4–8). The experimental details and data treating can be found in the Methods. Figure 1a presents a representative true stress-time curve of crosslinked PE melt under extension with strain rate of 3 s^−1^ at 172 °C as well as the selected two-dimensional (2D) wide-angle x-ray diffraction (WAXD) and small-angle x-ray scattering (SAXS) patterns. At the beginning of extension at low stress, only melt deformation is observed and no ordered structure appears (No. 1). At extension time of around 600 ms (No. 2), two symmetric streaks perpendicular to extensional direction show up in SAXS pattern, commonly regarded as scattering from shish or rod-like structure. Whilst absence of crystalline reflection in the simultaneous WAXD pattern indicates the non-crystalline nature of obtained shish. We name this non-crystalline shish as δ phase, which was assumed to be a vague kinetic state before and will be discussed later. Highly oriented crystal appears at around 770 ms (No. 3), evidenced by weak diffraction spots in WAXD pattern. Integrating 2D WAXD pattern into one-dimensional (1D) intensity curve, crystal forms of PE can be specified (Supplementary Fig. S1, x-ray wavelength is 0.103 nm). Single diffraction peak at 2θ of around 13.9° belongs to (100)_h_ reflection of hexagonal crystal (H-crystal), whilst two peaks simultaneously appearing at 2θ of around 14.4° (strong) and 15.5° (weak) correspond to (110)_o_ and (200)_o_ reflections of orthorhombic crystal (O-crystal), respectively. Under extension at 172 °C, the structural evolution is melt→non-crystalline δ→H-crystal, while no O-crystal is induced. Here we define the onset stress for observing characteristic WAXD or SAXS signal as the critical stress for relevant structure formation.

### Non-equilibrium crystallization phase diagram

By extracting critical stresses for the formations of δ phase, H- and O-crystals at different temperatures, we construct the non-equilibrium thermodynamic phase diagram for FIC of PE in stress-temperature space. The phase diagram composes of four phase regions, namely melt (L), non-crystalline shish (δ), H-crystal (H) and O-crystal (O), as shown in Fig. 1b (note the symbols are experimental points). The boundary between different phase regions is the critical stress for relevant structure transitions at different temperatures. The stress value may vary with molecular parameters, but the overall shape of the diagram is expected to be similar. The δ-O-H and the L-δ-O triple points are located at around (148 °C, 3.3 MPa) and (130 °C, 2.2 MPa), respectively. Note that the sample has been crystallized partially before extension at temperature below 120 °C, where the critical stress for δ phase in the diagram is just related to the onset of streak signal. The phase boundaries show different slopes in the diagram. Increasing stress *σ* leads to a sharp increase of L→δ transition temperature *T*_*L−δ*_ with *dT*_*L−δ*_/*dσ* of about 22 °C/MPa, while *dT*_*O−H*_/*dσ* shows a weak negative value of about −5.8 °C/MPa with stress exceeding the δ-O-H triple point. *dT*_*δ−H*_/*dσ* starts with a value of about 10 °C/MPa at low stress region, which keeps decrease with stress increase and reaches near zero at sample fracture point. Whether *dT*_*δ−H*_/*dσ* also has a negative value cannot be answered now, which requires future validation with specially designed sample that can endure larger stress. Interestingly, the negative value of *dT*_*O−H*_/*dσ* corresponds exactly to conversion from high-ordered O-crystal to conformational disordered H-crystal. Unlike the conventional picture of flow-induced ordering in polymer melt, flow-induced disordering is observed experimentally on the premise of applying large enough stress.

### Reversibility of structural transitions

**Figure 2:**
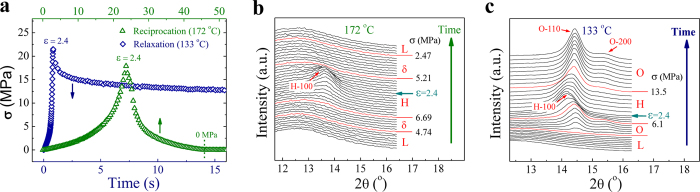
Evolutions of 1D WAXD curves on the processes of stress increase and decrease. (**a**) True stress-time curves of the reciprocating extension (172 °C) and the stress relaxation experiments (133 °C), respectively. The stress begins to decrease after reaching maximum strain (ε) of 2.4. (**b**) 1D WAXD curves of the reciprocating extension experiment at 172 °C. The marked red curves denote where structure forms or disappears and the horizontal arrow indicates the curve corresponding to the maximum strain of 2.4. The critical stresses for L→δ, δ→H, H→δ and δ→L transitions are 4.74, 6.69, 5.21 and 2.47 MPa, respectively. (**c**) 1D WAXD curves of the stress relaxation experiment at 133 °C. The critical stresses for O→H and H→O transitions are 6.1 and 13.5 MPa, respectively.

To verify the reversibility of flow-induced phase transitions and construct melting phase diagram during stress reduction, both reciprocating extension and stress relaxation experiments were performed (see experimental procedures in the Methods). The reciprocating extension experiment at 172 °C is selected as a representative for experiments above δ-O-H triple point, while the stress relaxation experiment at 133 °C is a representative for those below δ-O-H triple point. Figure 2a presents the corresponding true stress-time curves, where stress begins to decrease after strain reaches 2.4. For conciseness, we omit SAXS patterns (see Supplementary Fig. S2) and only present 1D WAXD curves. Figure 2b shows that, with the rise of stress under extension, structural evolution follows a path of L→δ→H and H-crystal reaches its maximum content at strain of 2.4. The critical stresses for observing δ and H phases are 4.74 and 6.69 MPa, respectively, close to the results of extension with strain rate of 3 s^−1^ in Fig. 1b (4.78 and 7.04 MPa). Note that the specific influence of strain rate on critical stress has been discussed in Supplementary Information combining with experimental results of non-crosslinked PE. Structure melting occurs when strain reduces in reciprocal extension, where H-crystal transforms back into δ phase at 5.21 MPa and the later further melts at lower stress of 2.47 MPa, demonstrating a reversed transition of H→δ→L. Noticeably, the critical stresses for H→δ and δ→L transitions are smaller than for their reversed δ→H (6.69 MPa) and L→δ (4.74 MPa) transitions during stress increase. Therefore stress induced transitions of L↔δ and δ↔H are two pairs of non-equilibrium thermodynamically reversible phase transitions with stress hysteresis. The same phenomenon is also observed in the reversible solid-to-solid transition of O↔H as shown in Fig. 2c. The critical stress for ordering process of H→O is 13.5 MPa, significantly larger than 6.1 MPa for disordering transition of O→H. The stress gap may serve as an indicator for nucleation barrier and we name it as “overstress”. The overstresses for the reversible transitions of L↔δ, δ↔H and H↔O are 2.27 (172 °C), 1.48 (172 °C) and 7.4 MPa (133 °C), respectively. Although H- and O-crystals have rather similar molecular packings^18^, the H→O transition still requires a large overstress.

### Non-equilibrium melting phase diagram

**Figure 3:**
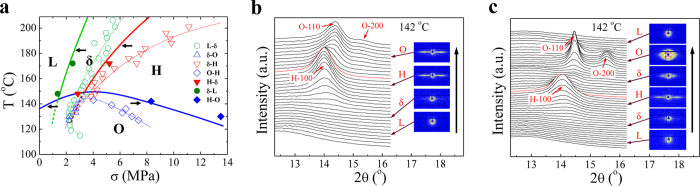
Melting phase diagram and structural and morphological evolutions in two different melting routes at 142 °C. (**a**) The non-equilibrium melting phase diagram of crosslinked PE under stress, where the solid points and thick lines correspond to the melting process. The short arrows indicate the shifting direction of melting lines with respect to crystallization lines. (**b**) 1D WAXD curves of the stress relaxation experiment at 142 °C. The up arrow indicates structural evolution direction and the red curve indicates where stress starts to decrease. Selected 2D SAXS patterns of different phases are shown as insertions. (**c**) 1D WAXD curves of the reciprocating extension experiment at 142 °C.

Based on the critical stresses for disordering and ordering in stress reduction, we construct the melting phase diagram in Fig. 3a. The short arrows indicate the shifting direction of melting lines with respect to crystallization lines (referring to Fig. 1b). Since the H→δ, δ→L and O→L are order-to-disorder transitions, the melting lines adopt a left-shift towards to lower stress. Whilst the solid-to-solid transition of H→O in stress reduction is an ordering process, whose boundary shows a right-shift to higher stress. The melting point of O-crystal with zero stress is about 136 °C. Comparing to that in stress increase, the stress window of δ phase is enlarged above the melting line of O-crystal, as the overstress of L↔δ is larger than that of δ↔H. Below the melting line of O-crystal, δ phase may occur as a metastable phase, as indicated by dash lines in the diagram, where the transition process is more complicated.

The existence of metastable δ phase is demonstrated by comparing the two melting routes of stress relaxation (Fig. 3b) and reciprocating extension (Fig. 3c) experiments at 142 °C. Besides the time evolution of 1D WAXD curves, 2D SAXS patterns are also presented to exhibit the corresponding morphologies. Increasing stress during extension, phase transition follows a pathway of L→δ→O→H in both cases (O-crystal has a short lifetime), consistent with the crystallization diagram in Fig. 1b. In the stress relaxation experiment, after a fast initial dropdown stress relaxes slowly and keeps at a relatively high level (around 6.8 MPa) locating in the stable O-crystal region, during which H-crystal transforms into O-crystal directly (see Fig. 3b). Whilst with the reciprocating extension experiment in Fig. 3c, the melting process follows an unusual pathway of H→δ→O→L along with fast stress reduction. Instead of directly transforming into O-crystal, H-crystal first melts into δ phase, which implies the existence of a metastable δ phase region hidden inside the stable O-crystal region. Further reducing stress or increasing time, O-crystal is formed with δ phase as the precursor. Actually, weak crystal diffraction still can be observed in δ region during H→δ→O transition (see Fig. 3c), which may be explained by kinetic reasons: (i) there is residue of H-crystal; (ii) a small amount of H phase directly transform into O-crystal; (iii) a small amount of δ phase transform into O-crystal ahead of time due to its metastability. It’s hard to determine which one is the main reason, but the very low content of crystal seems unlikely to produce the strong streak signal in SAXS. Surprisingly, the metastable δ phase appearing in reciprocating extension experiment is even observed at temperature down to 123 °C, far below the melting point (136 °C) at quiescent condition. The result not only confirms the existence of metastable δ phase region, but also indicates interesting kinetic competitions in the transitions among δ, H and O phases due to the stress hysteresis in ordering and disordering. H→δ transition is a fast melting process with low energy barrier, while H→O transition is a slow ordering process requiring a large overstress. Thus H-crystal kinetically favors “melting” into δ phase rather than “crystallizing” into O-crystal when stress is rapidly reduced even though the molecular packings in H- and O-crystals only have minor difference.

As demonstrated by 2D SAXS patterns in Fig. 3b,c, the morphologies of O-crystals obtained through two melting routes are obviously different. Only two streaks perpendicular to extensional direction are observed in the stress relaxation (see Fig. 3b), while additional to the streaks two elliptical scattering signals appear parallel to extensional direction in the reciprocal extension (see Fig. 3c). The corresponding crystalline morphology for the former is regarded as rod-like crystal, while the latter is periodically arranged lamellar crystals. The interplay of non-equilibrium thermodynamic stabilities and kinetic competitions of different phases creates a rich pool of phase transition behaviors, resulting in diverse final structures and morphologies.

### Temperature-driven phase transitions

**Figure 4:**
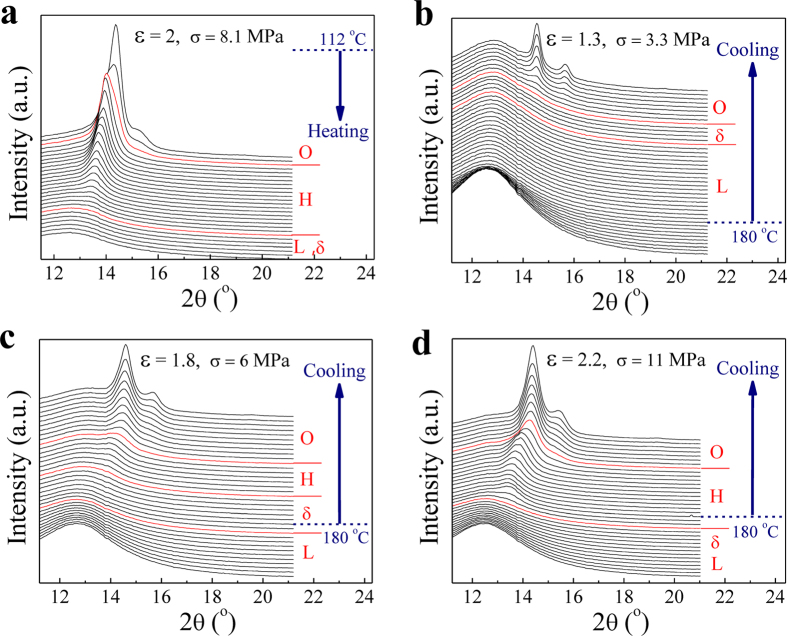
Evolutions of 1D WAXD curves during heating and cooling samples under fixed strain. (**a**) 1D WAXD curves during heating the sample from 112 °C under fixed strain of 2. The marked red curves denote where structure forms or disappears and the down arrow indicates the direction of increasing temperature. (**b–d**) 1D WAXD curves during cooling the samples from 180 °C under different strains of 1.3, 1.8 and 2.2, respectively. The short dash line denotes the starting point of cooling and the up arrow indicates the direction of decreasing temperature.

By performing heating and cooling procedures under fixed strain, the crystallization/melting phase diagrams are confirmed to be valid on predicting temperature-driven phase transition pathways. Figure 4a shows a representative of heating process, where the sample was first extended to strain of 2 at 112 °C and held for a long time until the stress was relatively stable (about 8.1 MPa). 1D WAXD curves clearly show O-crystal transforms into H-crystal with increasing temperature. After H-crystal melts completely, the streak signal still remains in SAXS pattern, indicating the independent appearance of δ phase. Thus the structural evolution follows a route of O→H→δ→L during heating. The phase transition pathways in cooling are checked with three experiments by stretching samples to different strains at 180 °C. Note stress reduction inevitably happens accompanied with temperature decrease because of stress relaxation at a fixed strain. Under strain of 1.3 (stress of 3.3 MPa), a transition of L→δ→O is observed with decreasing temperature (Fig. 4b). Cooling from δ phase at strain of 1.8 (stress of 6 MPa) leads to a pathway of δ→H→O (Fig. 4c), while starting with H-crystal at strain of 2.2 (stress of 11 MPa) H→O transition occurs (Fig. 4d). Although stress cannot be accurately linked with temperature due to the measuring difficulty, phase transition pathways during heating and cooling under fixed strain precisely fall into the predictions of the non-equilibrium crystallization/melting diagrams in Figs 1b and 3a (Supplementary Fig. S3).

### Interplanar spacing of H- and O-crystals

**Figure 5:**
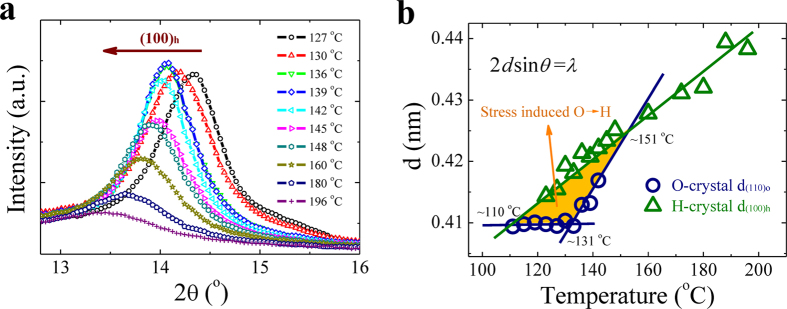
Interplanar spacing of H- and O-crystals. (**a**) 1D WAXD curves of H-crystal collected under extension just before samples facture. The diffraction peak of (100)_h_ plane shifts to lower 2θ with increasing temperature. (**b**) Interplanar spacing (d) of H-crystal (100)_h_ and O-crystal (110)_o_ planes as a function of temperature, respectively. Bragg equation (2*d* sin*θ* = λ) is used for calculation. The data of O-crystal come from the last WAXD patterns either before samples fracture or before its transformation to H-crystal. The yellow color marks the possible region for occurrence of O↔H transition driven by flow.

To understand details of flow-induced O↔H transition, the variation of lattice parameter for specific crystal is further investigated. The last 1D WAXD curves under extension before samples fracture are selected for comparison in Fig. 5a, which belong to H-crystal diffraction. The diffraction angle (2θ) of (100)_h_ plane presents continuous decrease from 14.35 to 13.45° as temperature increases from 127 to 196 °C. This reflects a lattice inflation of H-crystal that may be attributed to both thermal expansion and flow-induced additional conformation defects brought by raising temperature. By calculation with Bragg equation 2*d* sin*θ* = *λ* (*λ*, x-ray wave length), Fig. 5b presents the variations of H-crystal interplanar spacing *d*_(100)h_ and O-crystal *d*_(100)o_, respectively. Increasing extension temperature leads to a nearly linear increase of *d*_(100)h_ with a slop of 3.6 × 10^−4^ nm/°C. Whilst *d*_(100)o_ displays two different behaviors separated by 131 °C, which keeps almost constant below 131 °C but increases linearly with a slop of 7.1 × 10^−4^ nm/°C beyond 131 °C. As a result, *d*_(100)h_ and *d*_(110)o_ intersect at two temperature points of around 110 and 151 °C, respectively.

Note that the above three characteristic temperatures are significant for us to understand FIC in PE. First, flow-induced disordering of O-crystal starts to appear at 131 °C, which actually corresponds to the *α*_2_ mechanical relaxation of PE^19^. Such a disordering transition is deemed to arise from the onset of torsional vibrations of chain segments in crystal lattice and lead to entropy increase, as supported by dramatical increase of loss modulus^20^. Second, *d*_(110)o_ of conformation ordered O-crystal must be smaller than *d*_(100)h_ of conformation disordered H-crystal at a fixed temperature. In this aspect, H-crystal is impossible to form below 110 °C when *d*_(100)h_ is reduced to lower than *d*_(110)o_, regardless of stress. Similarly, O-crystal is also impossible to occur above 151 °C, which accords well with the highest point of stable O-crystal region in the crystalliztion phase diagram (Fig. 1b). At middle temperatures, both H- and O-crystals can be obtained as well as their mutual transformations determined by applied stress, as indicated by the yellow region in Fig. 5b.

## Discussion

With the aid of the non-equilibrium phase diagrams of flow-induced crystallization and melting, nearly all observations in FIC of PE can be well understood. One of the most controversial issues in crystallization under flow as well as at quiescent condition is the so-called non-crystalline pre-order^21^,^22^,^23^. By definition, pre-orders are transient structural intermediates inserted in the pathway from liquid to crystal, which are thought to be responsible on assisting nucleation of crystal through lowering down energy barrier. However, whether pre-order is a thermodynamic phase or a kinetic state remains unknown till current work. According to two experimental observations, (i) during crystallization, streaks in SAXS appear earlier than crystal diffractions in WAXD, (ii) during melting, SAXS streaks however disappear later than crystal diffractions, it was shown that δ is non-crystalline structure intermediate between amorphous melt and crystal. In terms of assisting crystal nucleation, δ structure indeed acts as “pre-order”. However, considering the physical basis for phase, δ structure may be an independent order rather than simply the pre-order for other phases. First, this δ can be observed solely without crystal diffractions during the whole extension process at temperature over 200 °C, which indicates that the existence of δ can be independent on formation of crystal. Second, the results of crystallization and melting in Figs 1b and 3a show that the direction and pathway of δ structural transitions, with other thermodynamic phases of amorphous melt, hexagonal phase and orthorhombic phase, are actually demonstrated by two state parameters of temperature and stress. The reversibility of transitions between state regions for δ state, depending on certain state parameters, is an important feature of phase. Thus, as suggested by the non-equilibrium crystallization and melting diagrams in Figs 1b and 3a, respectively, the δ phase defined in present work is a thermodynamic phase, which can be stable or metastable depending on the specific state determined by parameters of stress and temperature. In addition, the present x-ray data also show that δ phase has orientation and higher density with respect to the amorphous melt, but has no long-range order, which is similar to the ordering of liquid crystal.

The stress dependent reversible transitions among L, δ, H and O phases of PE result in unexpectedly rich pathways of FIC as demonstrated above. In a standard step-strain FIC experiment at temperature above L-δ-O triple point, δ phase can either nucleate from L phase during flow or melt back from O- or H-crystal during stress reduction, which may not be differentiable in a fast “short-term” flow. Below the melting line of O-crystal, the negative *dT*_*O−H*_/*dσ* and the hidden metastable δ phase region bring more choices for PE to crystallize. Owing to stress hysteresis for ordering and disordering or overstress we introduced, not only can the metastable δ phase serve as a transient intermediate order for nucleation of O-crystal from L phase during extension (L→δ→O), but also it can occur through H-crystal jumping over the stable O-crystal region during stress reduction (H→δ→O).

The non-equilibrium stress-temperature phase diagrams are intrinsically different from equilibrium pressure-temperature phase diagram of PE. Uniaxial stress of around 3 MPa is sufficient to induce the formation of H-crystal, while more than 300 MPa of high pressure is required^24^. The two orders gap essentially stems from stress-induced conformation ordering owing to long-chain nature of polymer^25^,^26^, which further couples with density^27^, while pressure drives density directly. Excessive stress can destroy the conformation order and induce conformation defects, making a negative value of *dT*_*O−H*_/*dσ*. This phenomenon may be analogue to pressure-induced disordering as observed experimentally in the crystalline polymer poly(4-methylpentene-1) which supports the speculation of reversing melting proposed by Tammann^28^,^29^. It demonstrates that the non-equilibrium process stimulated by flow is fundamentally different from equilibrium phase behaviors, where a rich source of physics is still waiting for us to dig out.

We would like to note two additional points relevant to the non-equilibrium phase diagrams. (i) The existence of δ phase at temperatures far above the equilibrium melting temperature may give new interpretation on non-linear rheological behaviors like strain hardening or softening, which have been interpreted with tube pressure or nematic interaction but without direct evidence^30^,^31^. These non-linear rheological phenomena may be originated from flow-induced ordering or density fluctuation. In this respect, the occurrence of δ phase at temperature range for rheological study can serve as the first experimental demo. (ii) The stress-temperature crystallization/melting diagrams are essentially the processing diagrams of polymer. The rich pathways of FIC provide a detailed roadmap for producing PE with designed structures and morphologies as well as final properties. Typical example can be found in explaining the so called “extrusion window” (a narrow temperature interval close to 150 °C) of reduced flow resistance existed in PE melt. As deeply studied by Keller *et al*.^32^, the formed H-crystal is demonstrated to be the structural origin of this special flow behavior, which accords well with the predication of the crystallization diagram in Fig. 1b. Taking the annual global PE consumption of 90 million metric tons (about one third of total polymer consumption), we envision that the processing phase diagram will bring a significant impact on polymer industry.

In summary, we have constructed the non-equilibrium phase diagrams of flow-induced crystallization and melting of PE, composing of melt, non-crystalline δ phase, H- and O-crystals. The non-crystalline δ is a thermodynamic phase rather than a kinetic state, which appears as a metastable transient pre-order for crystallization in stable O-crystal region. While beyond O-crystal region, δ behaves as an independent stable phase even at temperature up to 240 °C far above the quiescent equilibrium melting point of PE. The lattice parameters of O- and H-crystals under flow display different temperature dependences, which determines their formations and mutual transformations. The reversible transitions among the four phases with stress hysteresis create a rich pool of kinetic pathways for FIC, which are expected to serve as a detailed roadmap for precisely processing of PE with designed structures and morphologies.

## Methods

### Material

High density polyethylene (HDPE) was purchased from Sinopec Qilu Co. Ltd with number-average (M_n_) and weight-average (M_w_) molecular weights of 42 and 823 kg/mol, respectively. The raw HDPE granules were first molded evenly into plates with a thickness of 1 mm by a vulcanizing press at 190 °C and then cooled down to room temperature. The residual stress was eliminated by annealing treatment under vacuum at 90 °C for 20 h. After that the sample was exposed to a ^60^Co γ-ray radiation (located in USTC, Hefei, China) at 25 °C, where oxygen was isolated from sample in order to reduce peroxide radicals during the radiation process. The total absorbed dose was 100 KGy under radiation with a dose rate of 105 Gy/min for 16 h. The trapped free radicals in sample were further eliminated through annealing at 90 °C for 20 h under vacuum.

### Gel fraction characterization

The crosslinked PE was extracted fully in boiling xylene for 48 h with Soxhlet extractor. Then it was dried in a vacuum oven at 70 °C for 24 h until no decrease of weight. The calculation of gel faction was made gravimetrically from the weight of sample before and after extraction, which is 43.5% in this work. As comparison, it is 0% for non-crosslinked PE. Here the gel fraction can be also expressed as the crosslinked degree.

### Experimental procedures

A homemade two-drum extensional rheometer was employed to impose well defined flow field and thermal history on sample. As schematically drawn in Supplementary Fig. S4, the sample is secured to two geared drums by means of thin clamps. Stretching is realized by servo motor driving two geared drums to synchronously rotate in opposite directions, where Hencky strain is obtained. The sample length subjected to stretch is unchanged (equals to the distance *L*_*o*_ between two rotating axises), and the strain rate 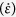 keeps constant as 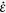 = 2*V*/*L*_*o*_ under a rotational velocity *V* of each drum. In experiments, each sample (30 × 20 × 1 mm^3^) was first heated to 200 °C and held for 10 min to erase thermal and mechanical history. Thereafter, it was rapidly cooled or heated to temperature *T*_c_, followed immediately by extension. A slow nitrogen gas flow was used as heating medium to homogenize temperature and prevent sample from degradation, which realized a temperature fluctuation within ±0.5 °C. The extension temperature covered a range from 111 to 240 °C. Torque was recorded in real-time during and after extension with a torque sensor to characterize the stress response. To investigate FIC, extension with strain rate of 3 s^−1^ until sample fracture was performed. Whilst the melting behaviors of ordered structures were studied through two protocols of stress reduction: (i) reciprocating extension by stretching the sample to a strain of 2.4 at 0.1 s^−1^ and subsequently decreasing the deformation to zero stress at the same strain rate, and (ii) stress relaxation under fixed strain of 2.4. The cooling experiments in Fig. 4b–d were realized by high-speed cool nitrogen gas blowing the sample with a cooling rate of about −10 °C/s after the cessation of flow.

### X-ray diffraction and scattering

Flow-induced structural evolution was detected by simultaneous WAXD and SAXS with a time-resolution of 50 ms at the BL19U2 station of Shanghai Synchrotron Radiation Facility (SSRF) and the 1W2A station of Beijing Synchrotron Radiation Facility (BSRF)^33^. X-ray wavelengths were λ = 0.103 and 0.154 nm at SSRF and BSRF, respectively. The Pilatus 1 M detector (1043 × 981 pixels and pixel size of 172 μm) was employed to collect 2D SAXS pattern and the Pilatus 300 K (487 × 619 pixels and pixel size of 172 μm) to collect 2D WAXD pattern. The sample-to-detector distances were calibrated to be 223 mm by yttrium sesquioxide (Y_2_O_3_) for WAXD, while 5850 mm by beef tendon for SAXS. WAXD and SAXS in current experiments cover a *q* range of 9.7–30.5 nm^−1^ and 0.049–0.76 nm^−1^, respectively. Fit2D software from European Synchrotron Radiation Facility was used to analyze the data which were corrected by subtracting contributions from the extensional rheometer and air.

## Additional Information

**How to cite this article**: Wang, Z. *et al*. The non-equilibrium phase diagrams of flow-induced crystallization and melting of polyethylene. *Sci. Rep*. **6**, 32968; doi: 10.1038/srep32968 (2016).

## Supplementary Material

Supplementary Information
