# Supplementary material for: The non-equilibrium phase diagrams of flow-induced crystallization and melting of polyethylene
Source: Sci Rep. 2016 Sep 9;6:32968. doi: 10.1038/srep32968 (PMC5016777; doi:10.1038/srep32968)
Supplement: Supplementary Information [file srep32968-s1.doc]

**Supplementary Information for**

**The** **non-equilibrium phase diagrams of flow-induced crystallization and melting of** **polyethylene**

Zhen Wang^1^, Jianzhu Ju^1^, Junsheng Yang^1^, Zhe Ma^2^, Dong Liu^1^, Kunpeng Cui^1^, Haoran Yang^1^, Jiarui Chang^1^, Ningdong Huang^1^, Liangbin Li^1,*^

^1^National Synchrotron Radiation Lab, CAS Key Laboratory of Soft Matter Chemistry, University of Science and Technology of China, Hefei, China. ^2^Tianjin Key Laboratory of Composite and Functional Materials, and School of Materials Science and Engineering, Tianjin University, Tianjin 300072, China.

*Email: [lbli@ustc.edu.cn](mailto:lbli@ustc.edu.cn) (L.L.)

**Supplementary Figures**


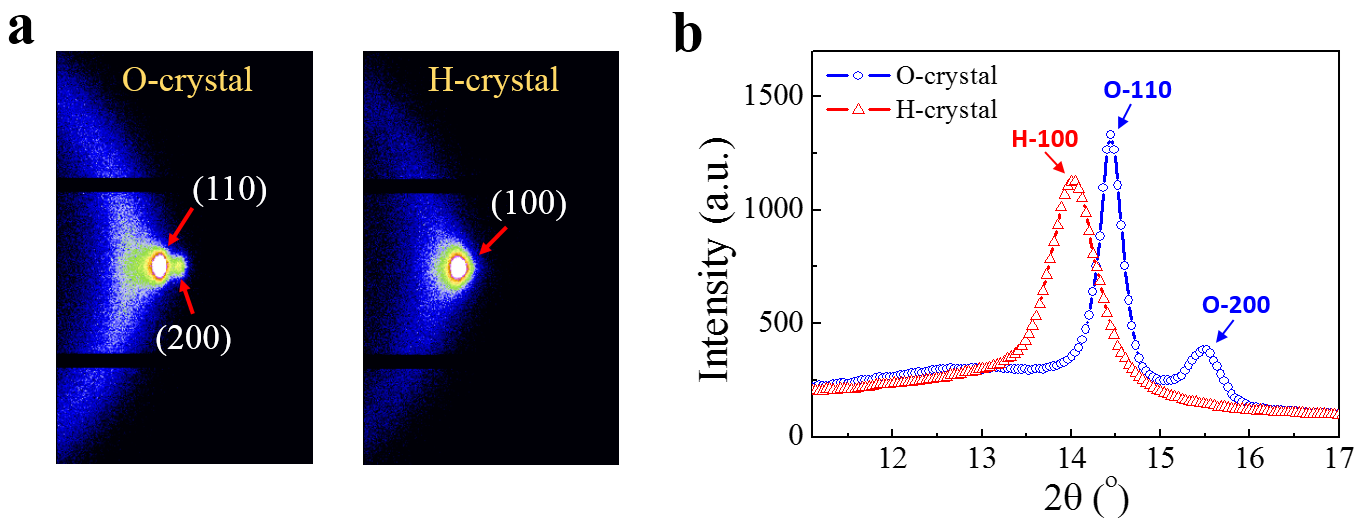


**Supplementary Figure S1 | Identification of crystal forms.** (**a**) 2D WAXD patterns of O- (left) and H-crystals (right) with extensional direction being vertical. (**b**) The corresponding 1D intensity curves.


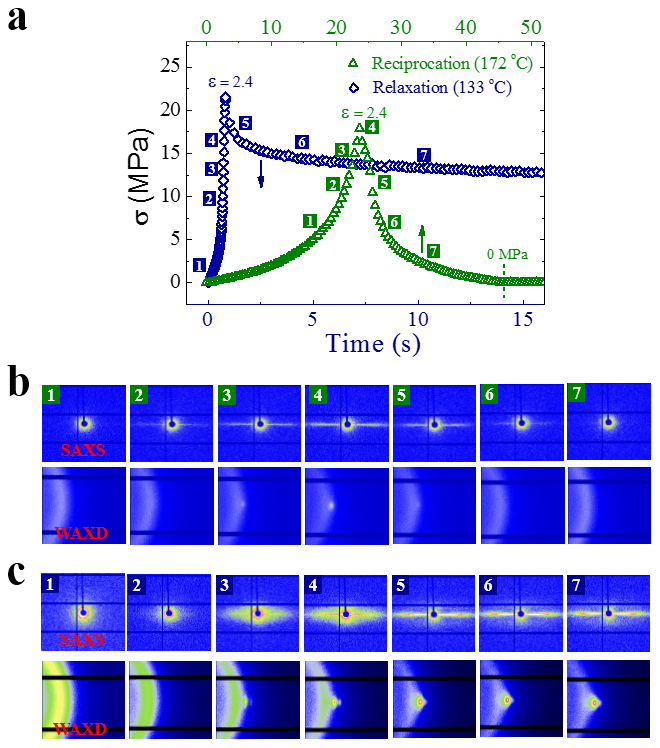


**Supplementary Figure S2 | Evolutions of 2D SAXS and WAXD patterns on the process of stress increase and decrease.** (**a**) True stress-time curves of the reciprocating extension (172 ^o^C) and the stress relaxation experiments (133 ^o^C), respectively. (**b**) Selected 2D SAXS and WAXD patterns of the reciprocating extension experiment at 172 ^o^C. (**c**) Selected 2D SAXS and WAXD patterns of the stress relaxation experiment at 133 ^o^C.


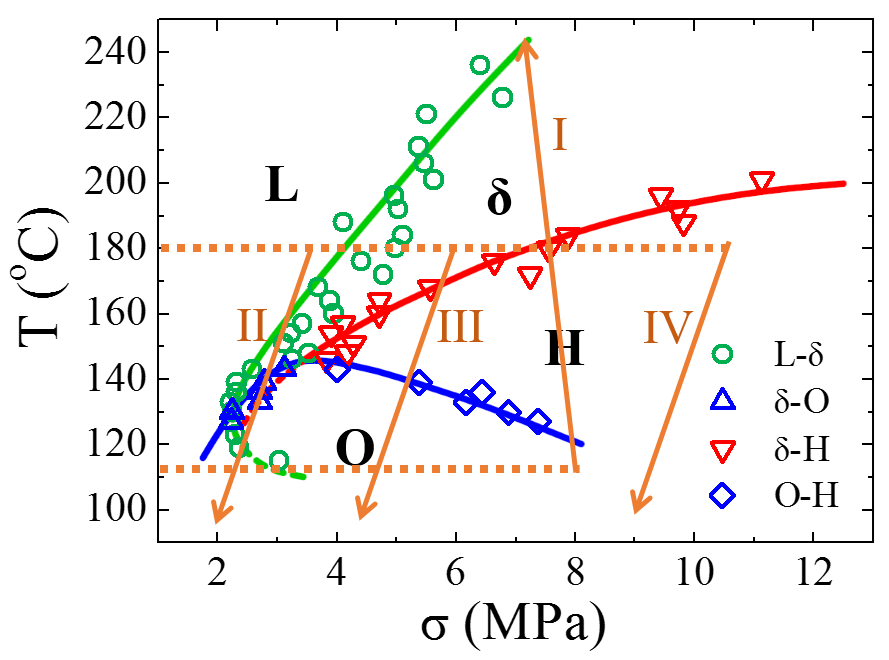


**Supplementary Figure S3 | Kinetic pathways of structural evolutions driven by heating and cooling samples at fixed strain.** Lines marked as I, II, III and IV summary the results in Fig. 4a,b,c,d in the paper, respectively.


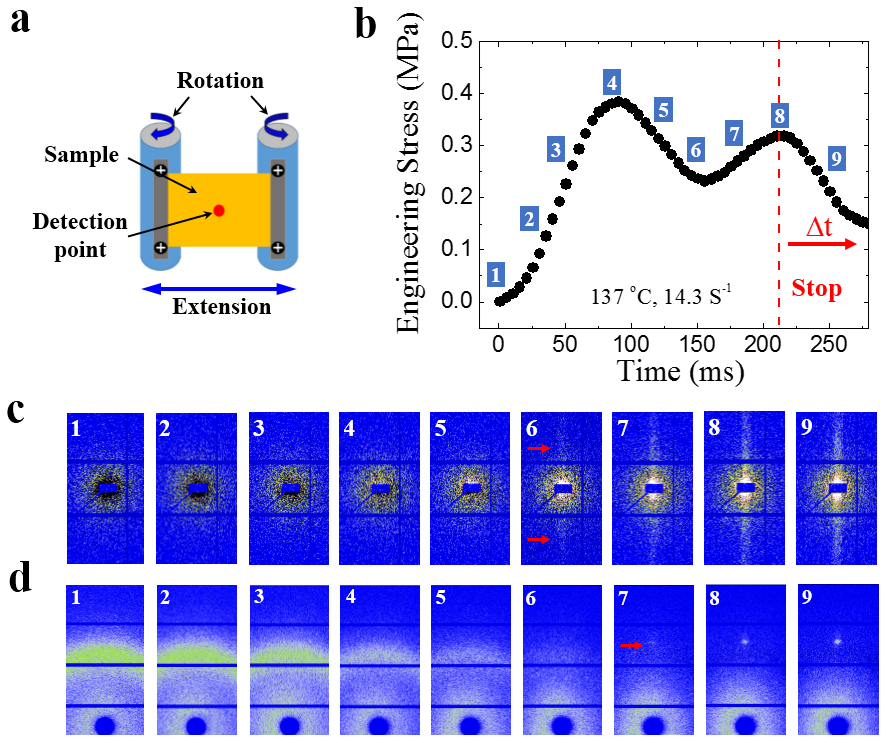


**Supplementary Figure S4 | FIC experiments on non-crosslinked HDPE melt.** (**a**) Schematic drawing of the homemade extensional rheometer. (**b**) Representative engineering stress-time curve under extension with strain rate of 14.3 s^-1^ at 137 ^o^C. The sample was stretched to strain of 3, followed by isothermal holding for a certain time ($\Delta t$). Collected (**c**) 2D SAXS and (**d**) WAXD patterns with a time interval of 30 ms corresponding to the numbered positions in (**b**). The arrows indicate streaks single in SAXS and crystal diffraction in WAXD, respectively. Note that the x-ray wavelength is 0.154 nm.


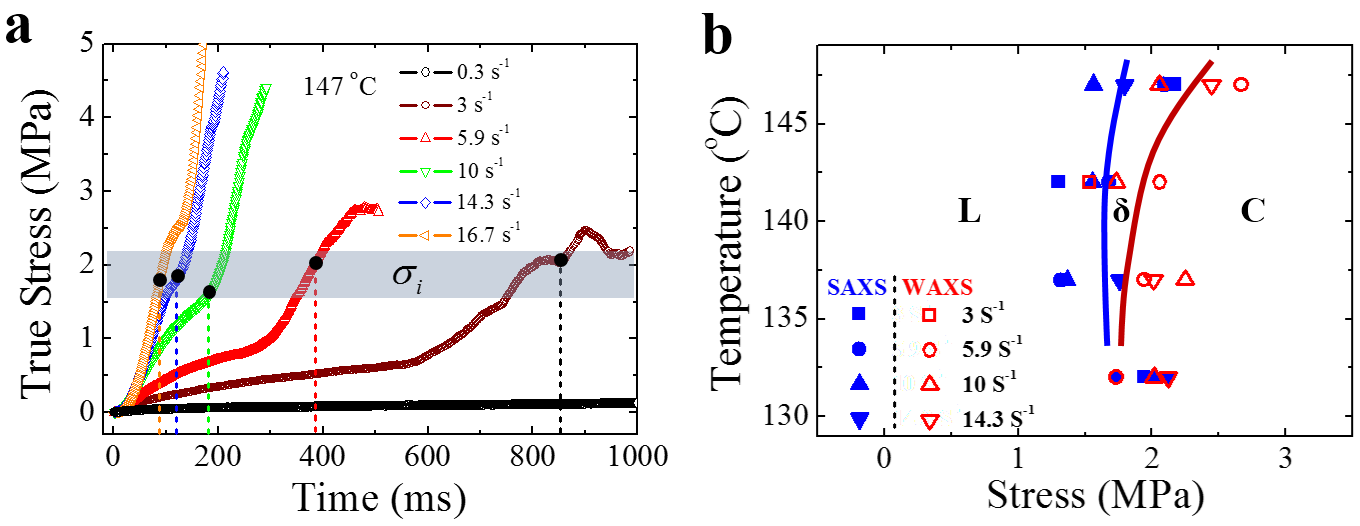


**Supplementary Figure S5 | Critical stresses for ordered structures formation.** (**a**) True stress-time curves under extension with different strain rates at 147 ^o^C. Black points indicate the onset stresses ($\sigma_{i}$) for observing δ phase. (**b**) The statistics of critical stresses over temperatures of 132, 137, 142, 147 ^o^C. With increasing stress, structure follows an evolution of L→δ→crystal (C).


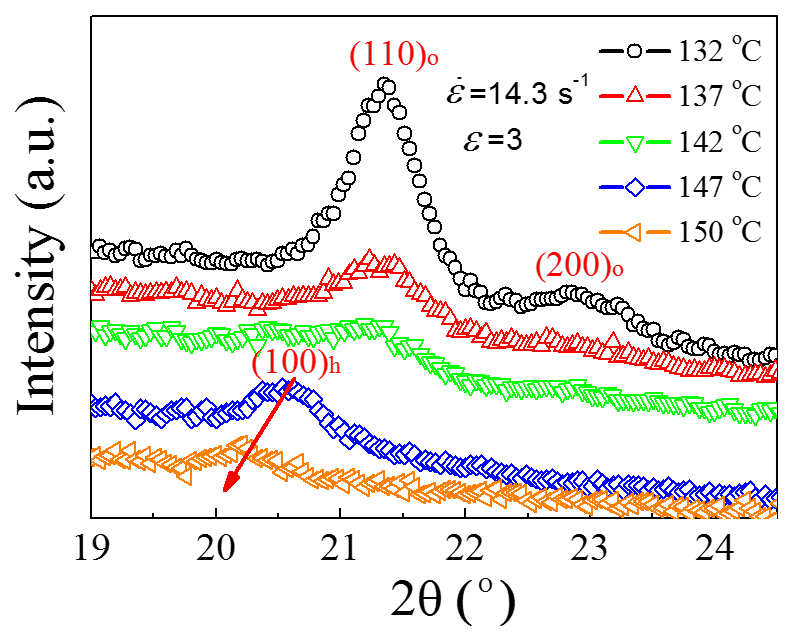


**Supplementary Figure S6 | Crystal forms induced by extension.** Increasing extension temperature from below to above the quiescent equilibrium melting point (141.4 ^o^C), the obtained crystal changes from O- to H-crystal. The red arrow indicates an obvious inflation of H-crystal even with a rather small temperature increase of 3 ^o^C.


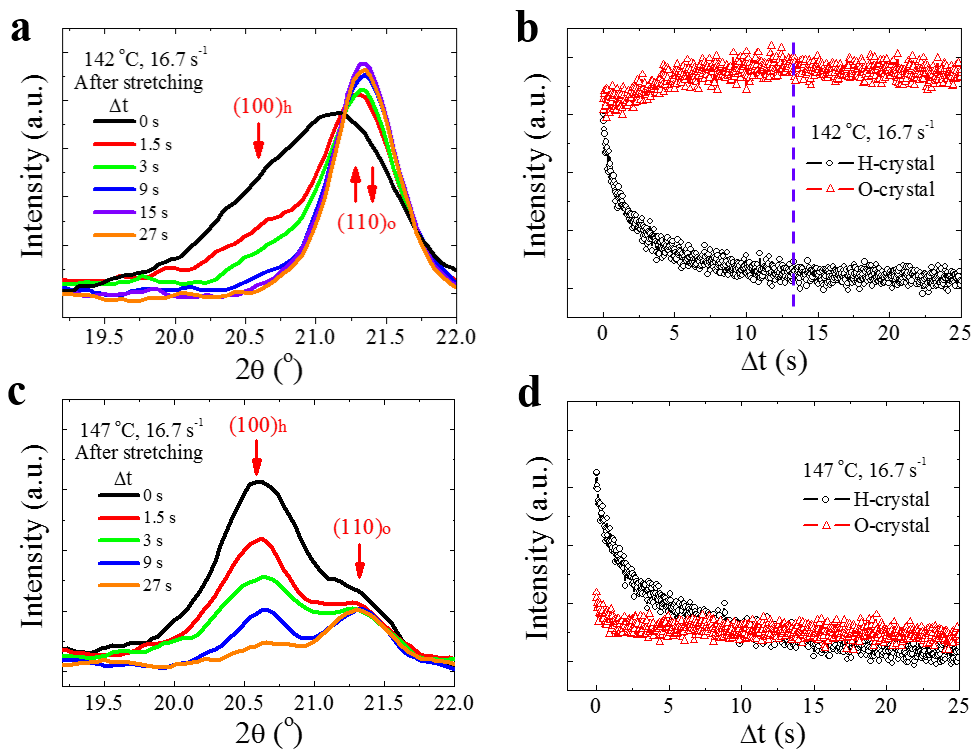


**Supplementary Figure S7 | Structural evolutions of HDPE during isothermal holding after extension.** Selected 1D WAXD curves and integrated diffraction intensities of O-crystal (110)_o_ and H-crystal (100)_h_ planes as a function of holding time at temperatures of (**a,b**) 142 and (**c,d**) 147 ^o^C, respectively. The arrows indicate the increase (up) or decrease (down) of diffraction intensity.


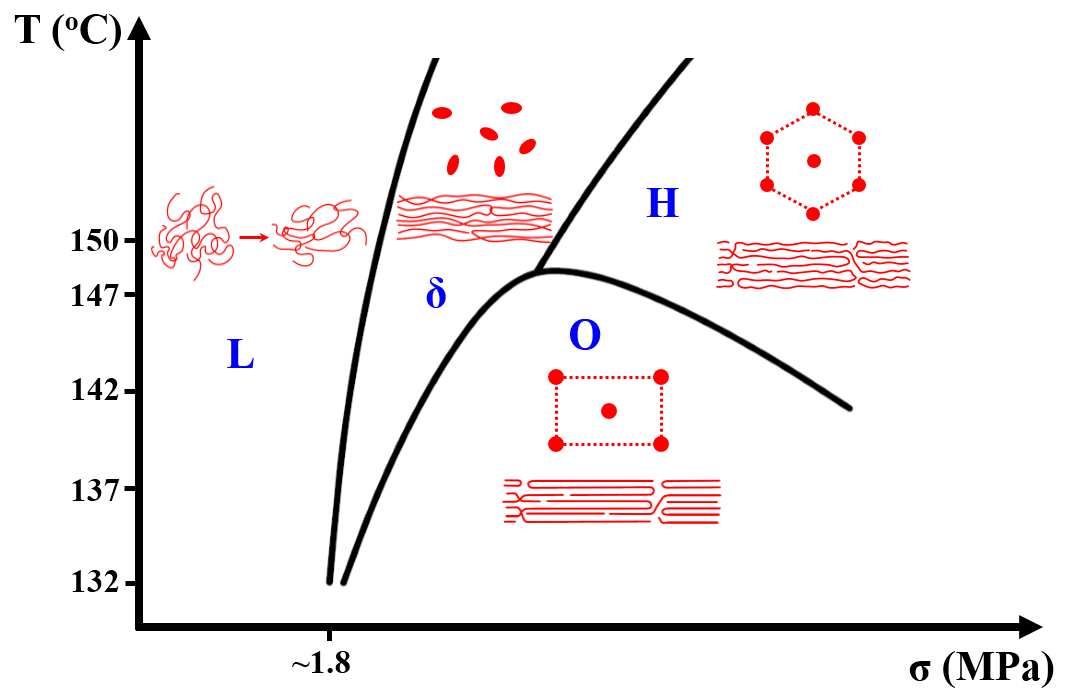


**Supplementary Figure S8 | Flow phase diagram of HDPE melt.** The inserted illustrations describe chain conformations of four phases. Melt (L) is amorphous, non-crystalline shish (δ) is orientated precursor, O-crystal (O) is crystal with all-trans chain conformation, and H-crystal (H) is crystal with disordered chain conformation.

**Supplementary Notes**

Figures S4,5,6,7,8 present experimental results of flow-induced crystallization in non-crosslinked HDPE with number-average (M_n_) and weight-average (M_w_) molecular weights of 42 and 823 kg/mol, respectively. With large enough strain rate (depending on temperature), the structural evolution of L→δ→crystal is reproduced. In past researches, H-crystal formed under flow was reported mainly limited to crosslinked or ultra-high molecular weight PE. Our current ultrafast x-ray scattering experiments confirm its occurrence in normal non-crosslinked PE melt (Supplementary Fig. S6). Comparing peak positions of (100)_h_ diffraction between 147 (2θ of 20.5 ^o^) and 150 ^o^C (2θ of 20.2 ^o^), the crystal inflation is further verified.

The variations of diffraction intensities from O- (increase) and H-crystals (decrease) at 142 ^o^C show a same time node of about 13 s (Supplementary Fig. S7a,b), which indicates a transition of H→O after extension. It can be well explained with the crystallization/melting phase diagrams in the paper. H-crystal is formed at stress beyond O-crystal region during extension, which would transform back into O-crystal once stress relaxes again into O-crystal region after extension. At 147 ^o^C (Supplementary Fig. S7c,d), no H→O transition is attributed to the rather narrow stress window for O-crystal. Based on experimental data in stretching non-crosslinked HDPE melt, we construct the corresponding flow diagram (Supplementary Fig. S8) which has a similar shape as that in crosslinked PE in the paper. Note the critical stress may show some difference due to their different molecular parameters.

Similar to stretching crosslinked PE in the paper, the stress control mechanism for structure formation is further demonstrated. As shown in Fig. S5, the onset stress for observing δ phase in non-crosslinked PE keeps the same level (about 1.9 MPa) for different strain rates (from 3 to 16.7 s^-1^) at 147 ^o^C. This demonstrates an independence of critical stress on strain rate at high temperature (supercooling $\Delta T$>0), which is also a reason why stress is selected as the flow parameter. But at low temperature ($\Delta T$<0), promotion of supercooling on nucleation cannot be ignored under flow, where lower strain rate would provide longer time for nucleation. In this case, L→O and δ→O are expected to depend on strain rate since L and δ are metastable in stable O phase region. While the critical stresses for L→δ and O→H may have no or weak dependence on strain rate, because the formations of δ and H rely mainly on applied flow (chain deformation or stress) rather than supercooling.
